# Human Respiratory Syncytial Virus Memphis 37 Grown in HEp-2 Cells Causes more Severe Disease in Lambs than Virus Grown in Vero Cells

**DOI:** 10.3390/v5112881

**Published:** 2013-11-22

**Authors:** Rachel J. Derscheid, Albert van Geelen, Jodi L. McGill, Jack M. Gallup, Tomas Cihlar, Randy E. Sacco, Mark R. Ackermann

**Affiliations:** 1 Department of Veterinary Pathology Iowa State University College of Veterinary Medicine, 1600 S. 16th St., Ames, IA 50011-1250, USA; E-Mails: avg@iastate.edu (A.G.); eag@iastate.edu (J.M.G.); 2 Ruminant Diseases and Immunology Research Unit, National Animal Disease Center/USDA/ARS 1920 Dayton Ave., Ames, IA 50010, USA; E-Mails: Jodi.McGill@ars.usda.gov (J.L.M.); Randy.Sacco@ars.usda.gov (R.E.S.); 3 Gilead Sciences, Inc., 333 Lakeside Drive, Foster City, CA 94404, USA; E-Mail: tomas.cihlar@gilead.com

**Keywords:** G protein, HEp-2, Infant, lamb, lung, respiratory syncytial virus (RSV), Vero

## Abstract

Respiratory syncytial virus (RSV) is the most common cause of bronchiolitis in infants and young children. A small percentage of these individuals develop severe and even fatal disease. To better understand the pathogenesis of severe disease and develop therapies unique to the less-developed infant immune system, a model of infant disease is needed. The neonatal lamb pulmonary development and physiology is similar to that of infants, and sheep are susceptible to ovine, bovine, or human strains of RSV. RSV grown in Vero (African green monkey) cells has a truncated attachment G glycoprotein as compared to that grown in HEp-2 cells. We hypothesized that the virus grown in HEp-2 cells would cause more severe clinical symptoms and cause more severe pathology. To confirm the hypothesis, lambs were inoculated simultaneously by two different delivery methods (intranasal and nebulized inoculation) with either Vero-grown or HEp-2-grown RSV Memphis 37 (M37) strain of virus to compare viral infection and disease symptoms. Lambs infected with HEp-2 cell-derived virus by either intranasal or nebulization inoculation had significantly higher levels of viral RNA in lungs as well as greater clinical disease including both gross and histopathologic lesions compared to lambs similarly inoculated with Vero-grown virus. Thus, our results provide convincing *in vivo* evidence for differences in viral infectivity that corroborate previous *in vitro* mechanistic studies demonstrating differences in the G glycoprotein expression by RSV grown in Vero cells.

## 1. Introduction

Respiratory syncytial virus (RSV) is a *Paramyxovirus* in the subfamily *Pneumovirinae* that is present worldwide, causing a range of respiratory disease in infants to the elderly [1,2,3,4]. Clinical manifestations primarily occur in infants, young children, elderly, or the immunocompromised, and the greatest morbidity and mortality occurs in infants and young children with an estimated 33.8 million cases per year of RSV lower respiratory disease in children under 5 years of age [1,3,4]. This population has a unique immune system that plays an integral role in disease progression; however, most animal models of disease are in adults.

Lambs have a less-developed immune system than adult sheep and similar lung development and structure to infants [5]. While rodents, the primary RSV-model species, demonstrate post-natal pulmonary alveolarization (the final stage of lung development), alveolarization in lungs of lambs begins prenatally as in humans [6,7]. Lung epithelium plays an active role in immunity [8] and the degree of maturation at the time of initial RSV infection is important to both in the increased morbidity and severity of disease in children. Also, instead of a simple bifurcation into two lung lobes, sheep have dichotomous branching into multiple lobes which is important in viral inoculation, delivery of possible therapies, and respiratory function. In addition, sheep are naturally susceptible to ovine and bovine strains of RSV and experimentally develop disease when inoculated with bovine RSV (bRSV) or human RSV (hRSV) [5,9,10]. These features make neonatal lambs a very attractive model for RSV infection in infants.

The G glycoprotein is a transmembrane protein embedded in the viral envelope and also occurs as a secreted protein [11,12,13,14]. The G protein is heavily glycosylated through post-translational modification in the endoplasmic reticulum and Golgi apparatus [15,16]. G protein attachment by Paramyxovirinae viruses to host cell membrane causes a structural change in the fusion protein (F), allowing the virus to incorporate its envelope into the host cell membrane [17]. The secreted form of the viral glycoprotein is involved in immune modulation and evasion [11,18]. While not essential for viral replication or infection, virus lacking G protein is attenuated [19,20]. HRSV will replicate in Vero cells; however, RSV grown in Vero cells produces a truncated attachment G glycoprotein that is approximately 55kDa rather than 90kDa [21]. Early *in vitro* studies that identified the G protein reported different sizes, likely due to the use of African green monkey cell lines versus other cell lines [13,22]. Ultrastructurally, RSV viral inclusions of Vero cells differ from those in HeLa or other cell lines, and syncytia are reportedly fewer [23,24].

Based on *in vitro* studies comparing Vero-grown RSV to virus grown in other cell lines, we hypothesized that HEp-2-grown RSV would cause more severe disease as evidenced by clinical disease, gross and histologic lesions, and viral replication [21]. This was tested by infecting neonatal lambs through two different inoculation routes with virus grown in each of the two cell types.

## 2. Methods

### 2.1. Experimental Design

The studies use Memphis 37 RSV strain that was provided by Meridian BioSciences, Inc. (Memphis, TN, USA) at 6 passages on Vero cells and then again passaged in our laboratory on either Vero or HEp-2 cells for two additional passes for preparation of inoculum. Briefly, one T-75 flask (Techno Plastic Products, Sigma, MO, USA) with HEp-2 cells at approximately 80% confluence was infected at an MOI of 0.5 and virus was harvested after 36 h. With this virus 2 × T-300 flasks with Vero cells at 80%–90% confluence and 2 × T-300 flasks with HEp-2 cells at 80% confluence were infected at an MOI between 0.5 and 1. Two to three days later, when virtually all cells were infected (exhibiting numerous syncytia), but still attached, the virus was harvested by scraping the cells from the flasks and clarifying the supernatant at 2500 × g. Sucrose was added to 8% and the virus stock was frozen at −80 °C and titered for infectivity on HEp-2 cells. RSV M37 is a wild type RSV-A first isolated from infected humans and used in human clinical studies [25]. The amount and dose of virus used in these studies is similar to those from our previous work with RSV A2 strain [10].

Neonatal lambs (2–5 days of age) were randomly assigned to four groups, a control group (*n* = 4), intranasally-inoculated Vero-grown (INV) M37 (*n* = 4), intranasally-inoculated HEp-2-grown (INH) M37 (*n* = 4), nebulized Vero-grown (NeV) M37 (*n* = 2), or nebulized HEp-2-grown (NeH) (*n* = 5). Each intranasally-inoculated lamb received 2 mL/nostril of 1 × 10^7^ pfu/mL in DMEM inoculum administered via a syringe attached to a mucosal atomization device (Wolfe Tory Medical, Inc., Salt Lake City, UT, USA). Control lambs were inoculated intranasally with 2 mL/nostril cell growth media. Lung tissue was collected from all lambs at six days post-inoculation as this is the timepoint of peak viral lesions [5,9,10]. Because NeV lambs had the potential to have reduced lesions (according to our hypothesis), additional lambs (*n* = 4) were similarly nebulized with Vero-grown virus and tissues were collected at 3 (*n* = 2) and 14 (*n* = 2) days post-inoculation (p.i.) and used to confirm that disease/lesions did not occur outside the 6-day timepoint. Each nebulized lamb received 3 mL of 1 × 10^6^ pfu/mL in DMEM for 10 min using a jet Nebulizer (VixOne) at 32 PSI (Philips Respironics Air Compressor, Andover, MA, USA) attached to a conical mask with an oval gasket in which the nose and mouth of the lamb was inserted. All lambs received daily antibiotics (Ceftiofur, 1–2 mg/kg, intramuscular) to prevent secondary bacterial infection. Lambs were monitored daily for clinical signs of respiratory disease and overall health. Clinical features were recorded daily and lung tissues were collected six days p.i. Animals were euthanized by sodium pentobarbital. Animal use and experimental procedures were approved by Iowa State University’s Animal Care and Use Committee. 

### 2.2. Western Blotting for G Protein

M37 was passed in Vero or HEp-2 cells twice as described above, then cell pellets from mock or infected Vero and HEp-2 cells were solubilized with commercial cell lysis buffer (BD Pharmingen, San Jose, CA, USA) containing a 1X concentration of cOmplete Mini Protease Inhibitor Cocktail with EDTA (Roche, Indianapolis, IN, USA). Protein concentrations were determined using the BCA Protein Assay kit per manufacturers’ instructions (Pierce, Rockford, IL, USA). Samples were reduced in Laemmli sample buffer with 5% β-mercaptoethanol and heated to 70 °C for 5 min, then 20 μg of sample were loaded into a 10% polyacrylamide gel containing sodium dodecyl sulfate and separated by electrophoresis. Proteins were transferred to nitrocellulose membranes using the iBlot Gel Transfer system (Invitrogen, Carlsbad, CA, USA) and the membranes were blocked for 30 min using StartingBlock Blocking Buffer (Thermo Scientific, Waltham, MA, USA). Membranes were probed with primary mouse anti-RSV G-protein monoclonal antibody (Clone 131/2G, Millipore) overnight at 4 °C in Tris buffered saline containing 0.1% Tween-20 (TBST) and 5% nonfat milk. Membranes were washed for 1 h in TBST with 5 or more buffer changes, then incubated for 1 h at room temperature in 5% nonfat milk-TBST with horseradish peroxidase (HRP)-labeled goat anti-mouse (Jackson ImmunoResearch). After washing, membranes were exposed to SuperSignal West Dura Extended Duration Substrate or SuperSignal West Femto Chemiluminescent Substrate (both from Pierce) and exposed to film. Protein sizes were estimated by comparing to Benchmark Prestained Protein Ladder (Invitrogen).

### 2.3. Post-Mortem

After euthanasia the thorax was opened, lungs were removed, and gross lesions were scored and photographed *in vivo* and *ex vivo*. Tissue samples were collected uniformly from all animals. Briefly, multiple samples from each lobe were snap-frozen in liquid nitrogen for RNA isolation and one-step reverse transcription quantitative polymerase chain reaction (RT-qPCR), two samples from each lobe were placed in tissue cassettes and put in 10% neutral-buffered formalin for histological and immunohistochemical analysis by a board-certified veterinary pathologist (Ackermann). 

### 2.4. One-Step Reverse Transcription Quantitative PCR for RSV and Cytokine mRNA Levels

Whole vials of right and left cranial, right and left middle, and accessory lobes were homogenized in TRIzol (Invitrogen, Carlsbad, CA, USA), then pooled for each animal to create a composite slurry. RNA isolation continued per manufacturer’s instructions (Invitrogen), followed by DNase treatment (Ambion, TURBO DNase, Austin, TX, USA), then diluted 1:10 with a combination of RNaseOUT (Invitrogen) and nuclease-free water (Invitrogen). Spectrophotometry (NanoDrop, Thermo Scientific) was used to assess each sample for purity and total RNA. Agilent Bioanalyzer 2100 analysis of RNA routinely showed RIN values of 8.0 or higher. Hydrolysis probe-based one-step RT-qPCR was carried out using One-Step Fast qRT-PCR Kit master mix (Quanta, BioScience, Gaithersburg, MD, USA) in a GeneAmp 5700 Sequence Detection System (Applied Biosystems, Carlsbad, CA, USA) employing PREXCEL-Q for all set up calculations [26]. Primer and probe sequences (Table 1) have been previously used in our lab. All sequences were generated using ABI Primer Express 2.0 software. All samples were diluted to achieve a final RT-qPCR concentration of 0.784 ng/μL. Each sample was assessed in duplicate and each target gene amplification Cq converted to a relative quantity (rQ) based on the standard curve and using the following equation: rQ = 10^((Cq−b)/m)^, where Cq is the target quantification cycle, and b and m are the y-intercept and slope, respectively, from the Stock I-derived standard curve for each target [26]. The efficiency-corrected delta Cq (E_AMP_^Δ^^Cq^) method was employed for RT-qPCR quantification analysis.

**Table 1:** Primer and probe sequences used for real-time qPCR, 5’ to 3’.

**M37 hRSV**	Fwd:	GCTCTTAGCAAAGTCAAGTTGAACGA	**IFN γ**	Fwd:	TGGAGGACTTCAAAAGGCTGAT
	Rev:	TGCTCCGTTGGATGGTGTATT		Rev:	GATGGCTTTGCGCTGGAT
	Probe:	6FAM-ACACTCAACAAAGATCAACTTCTGTCATCCAGC		Probe:	6FAM-CAAATTCCGGTGGATGATCTGC-TAMRA
**CCSP**	Fwd:	CAG CCC TGA CGA AGA CAT GA	**TNF α**	Fwd:	CAACCTGGGACACCCAGAAT
	Rev:	GGG TGT CTA CCA GCG TCT TCA		Rev:	TCTCAAGGAACGTTGCGAAGT
	Probe:	6FAM-AGA GGC AAC AAG TCA G-MGBNFQ		Probe:	6FAM-CAAGGGCCAGGGTTCTTACCGGAA-TAMRA
**SP-A**	Fwd:	TGA CCC TTA TGC TCC TCT GGA T	**TGF β**	Fwd:	TGTGTTCGTCAGCTCTACATTGAC
	Rev:	GGG CTT CCA AGA CAA ACT TCC T		Rev:	TAGCCCTTGGGTTCGTGAAT
	Probe:	6FAM-TGG CTT CTG GCC TCG AGT GCG -TAMRA		Probe:	6FAM-TCCAGCCCAGGTCCTTCCGGA-TAMRA
**IL-6**	Fwd:	GCTGCTCCTGGTGATGACTTC	**MCP1 α**	Fwd:	GCTGTGATTTTCAAGACCATCCT
	Rev:	GGTGGTGTCATTTTTGAAATCTTCT		Rev:	GGCGTCCTGGACCCATTT
	Probe:	6FAM-CTTTCCCTACCCCGGGTCCCCTG-MBGNFQ		Probe:	6FAM-AAAGAGTTTTGTGCAGACCCCAACC-TAMRA
**IL-8**	Fwd:	TTCCAAGCTGGCTGTTGCT	**MIP1 α**	Fwd:	CAGCAGCCAGTGCTCCAA
	Rev:	TTGACAGAACTGCAGCTTCACA		Rev:	ACCTGCCGGCCTTTTTTG
	Probe:	6FAM-CCGCTTTCCTGCTCTCTGCAGCTC-TAMRA		Probe:	6FAM-CCTGGTGTCATCTTCCAGA-MGBNFQ
**IL-10**	Fwd:	GTCGGAAATGATCCAGTTTTACCT	**RANTES**	Fwd:	TGCTTCTGCCTCCCCATATG
	Rev:	GTCAGGCCCATGGTTCTCA		Rev:	GGGCGGGAGATATAGGCAAA
	Probe:	6FAM-AGGAGGTGATGCCACAGG-MGBNFQ		Probe:	6FAM-CACCACGCCCTGCT-MGBNFQ
**IFN β**	Fwd:	TGGTTCTCCTGCTGTGTTTCTC			
	Rev:	CGTTGTTGGAATCGAAGCAA			
	Probe:	6FAM-ACCACAGCTCTTTCCAGGAGCTACA-TAMRA			

### 2.5. Gross Lesion Evaluation and Scoring

After removal, percentage parenchymal involvement was estimated for each lung lobe by a board-certified veterinary pathologist (Ackermann). Percentages were converted to a scale using the following formula: 0% = 0, 1–9% = 1, 10–39% = 2, 40–69% = 3, 70–100% = 4. Group averages were calculated for the gross lesion score.

### 2.6. Histologic Evaluation and Scoring

Lung histologic score was determined by estimating percent consolidation followed by conversion to a consolidation scale used by our laboratory previously [10] by a board-certified veterinary pathologist (Ackermann): 0% consolidation = 0, 1–9% consolidation = 1, 10–39% consolidation = 2, 40–69% consolidation = 3, 70–100% consolidation = 4. Group averages were calculated for the alveolar consolidation score.

### 2.7. Immunohistochemical Detection of RSV Antigen

Immunohistochemistry for RSV antigen was performed on paraffin-embedded tissue as described previously [10] with the following variations: instead of Pronase E antigen retrieval, after final water rinse of slides, antigen retrieval was performed in TE, pH 9.0 with heat and pressure (Decloaking Chamber™ Plus, Biocare Medical, Concord, CA, USA) using the factory default program which lasted about 40 min, during which time the peak temperature of 125°C was reached in about 18 min, and after which the system cooled down to 80°C in another 22 min. Primary polyclonal goat anti-RSV antibody (BioDesign/Meridian, San Ramon, CA, USA) was applied for 2 h at room temperature at a dilution of 1:300 instead of 1:50 as previously described. Color was developed with Nova Red (Vector, Burlingame, CA, USA), counterstaining with Harris’ hematoxylin, after which slides were dehydrated and cover-slipped. Slides were then assessed for both the number of positive 20X fields (out of 20) for each slide as well as number of positive cells per 20X field by a board-certified veterinary pathologist (Ackermann). The number of positive cells per field was then given a score according to the following scale: 0 = 0, 1 = 1–10, 2 = 11–39, 3 = 40–99, 4 = >100.

### 2.8. Statistical Analysis

All analyses were performed using GraphPad Prism 5. All post-mortem data was assessed with one-way ANOVA followed by Tukey’s post-test. RT-qPCR data was log transformed for normality, if necessary. All clinical data was assessed with two-way ANOVA and cumulative weight change was additionally assessed with one-way ANOVA followed by Tukey’s post-test.

## 3. Results

### 3.1. G Protein Expression in RSV Memphis 37-infected Vero and HEp-2 Cells

**Figure 1:**
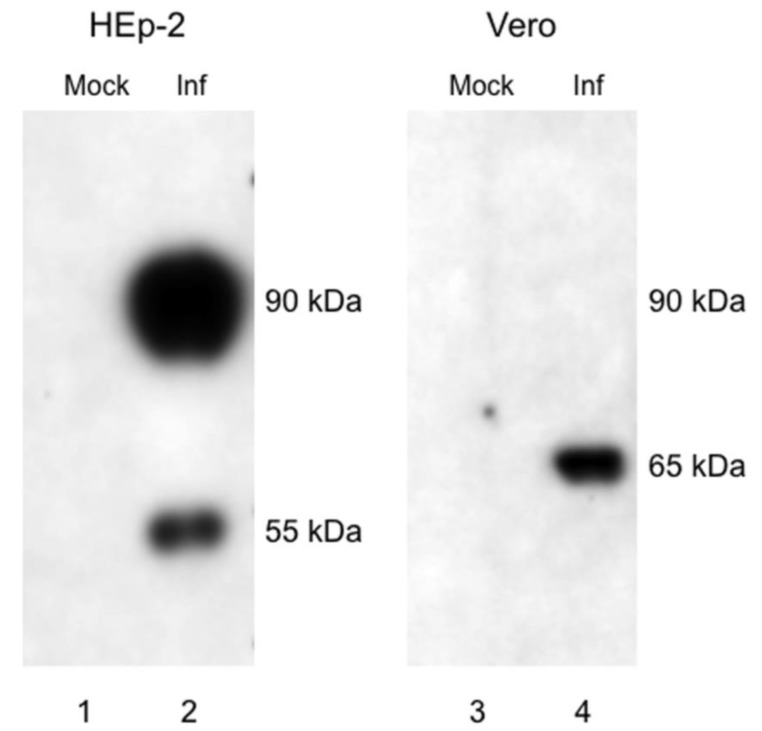
G glycoprotein expression RSV Memphis 37-infected Vero cells compared to infected HEp-2 cells. M37 RSV virus was grown for 2 passages in HEp-2 or Vero cells. Equivalent concentrations of whole cell lysate from mock or infected cells were then analyzed by western blot for expression of the RSV G protein. The majority of the G protein produced by RSV grown in HEp-2 cells was the 90 kDa form, with a minor band representing the 55 kDa form. In contrast, RSV grown in Vero cells produced undetectable levels of the 90 kDa form of the G protein, and instead produced a truncated 65 kDa form. Results are representative of three independent experiments.

Previously, it was shown that virions produced from RSV A2- or Long-infected Vero cells displayed primarily a smaller truncated form of approximately 55 kDa, whereas the 90 kDa form was predominately detected in virions from infected HEp-2 cells [21]. To determine whether similar results are obtained when Vero and HEp-2 cells are infected with RSV Memphis 37 strain, we performed western blot analyses by electrophoresing cell lysates and probing with a monoclonal antibody that recognizes RSV G protein. As shown in Figure 1, a major 90 kDa band and a minor 55 kDa band were observed in HEp-2-infected cells, whereas a smaller intermediate form of the G protein predominated in Vero cells. It should be noted that the intermediate form of the G protein seen in the present study using RSV Memphis 37 infection of Vero cells had a deduced molecular mass that was slightly larger (65 kDa) than in a previously published report (55 kDa) by Kwilas *et al.* [21]. However, the molecular mass of the truncated G protein from RSV-infected cell lines and virions has ranged from 45 kDa to 60 kDa in several studies [27,28,29]. Several potential explanations for the observed size variation in the intermediate form of the G protein from infected Vero cells include: inherent difficulties in determining molecular mass of heavily glycosylated molecules; possible variation in electrophoretic mobility of molecular mass standards; or differential processing of the RSV Memphis 37 G protein in Vero cells.

### 3.2. Clinical and Post-Mortem Findings

The NeV lambs had a significantly lower respiratory rate than the InV group on days 3 and 6, and NeV lambs had significantly lower respiratory rate than NeH lambs on the day of inoculation (day 0). There were no significant differences in body temperature or weight gain over the course of the study. Of the two NeV lambs, one had a mild cough and mild wheeze, though no gross lesions. There was no significant difference from uninfected control lambs in clinical symptoms.

**Figure 2:**
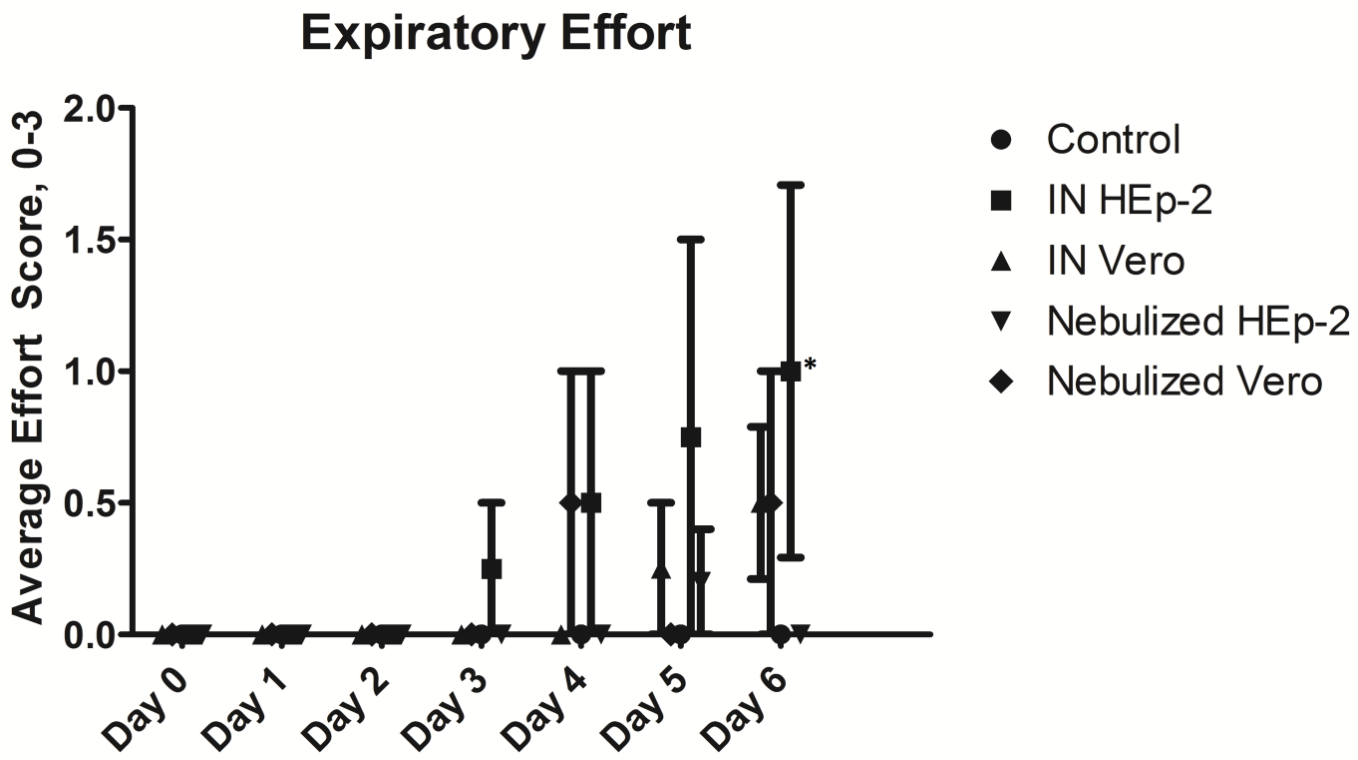
Daily clinical expiratory effort score, 0–3. Expiratory effort (e.g., forced expiratory effort; abdominal effort) was scored by: 0 = no expiratory effort; 1 = earliest detection of increased expiratory effort; 2 = moderate effort, observed without picking up lamb; 3 = hard abdominal effort with nostril flaring. On day 6 p.i. the intranasally-inoculated HEp-2-grown virus group had a significantly higher clinical expiratory effort score, *p* < 0.05 designated by an asterisk (*) compared to control lambs. Control *n* = 4, Intranasal (IN) HEp-2 *n* = 4, Intranasal (IN) Vero *n* = 4, Nebulized HEp-2 *n* = 5, Nebulized Vero *n* = 2.

**Figure 3:**
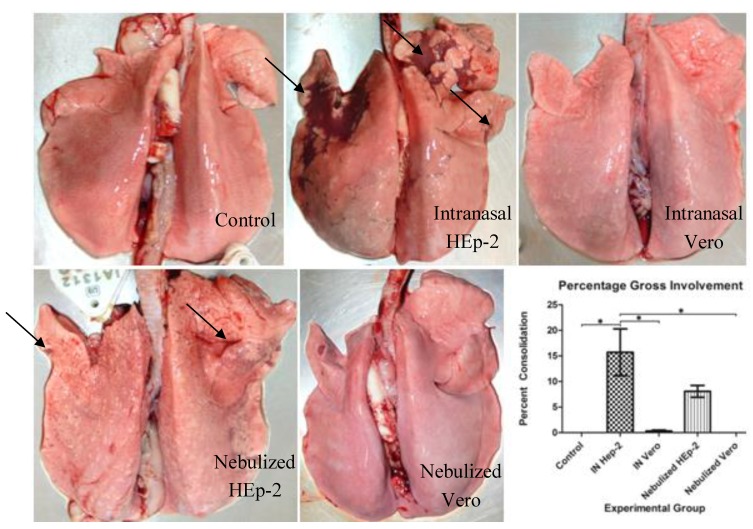
Gross lesions of infected lambs. Arrows indicate viral lesions characterized by reddened regions of consolidation. The intranasal HEp-2 (INH) group had significantly more gross involvement than control, intranasal Vero (INV), or nebulized Vero (NeV) groups.

Two of four INH lambs developed increased expiratory effort (forced expiration) with one of the lambs progressing to a grade 3 with clinical illness as evidenced by lethargy. The average expiratory effort scores had much variability but trended toward an increase and a significantly higher score for the IN HEp-2 group compared to control on day 6 (Figure 2). These two clinically symptomatic lambs had significant gross lesions characterized by large, well-delineated areas of dark red to red-gray discoloration and associated depressed parenchyma (atelectasis); a third lamb had very small, punctate areas of similar discoloration and the last lamb had no gross changes. These affected a larger portion of the cranial and middle lobes than the caudal or accessory lobes. Two of four INV lambs developed a grade 1 wheeze but the group wheeze score was never significantly different from control. These two lambs had small but distinct areas of slightly depressed dark red-gray discoloration. Of the five NeH lambs, one developed a grade 1 wheeze and one lamb developed a mild cough. All five of these lambs had dark red to red-gray multifocal, pinpoint to confluent foci of consolidation in most if not all lung lobes. The average gross lesion percentage score of the INH group was significantly higher than control lambs or INV lambs (Figure 3). Lambs inoculated with Vero-grown virus and euthanized at 3 and 14 days p.i. lacked significant clinical signs.

### 3.3. Histopathology Findings and Scores

**Figure 4:**
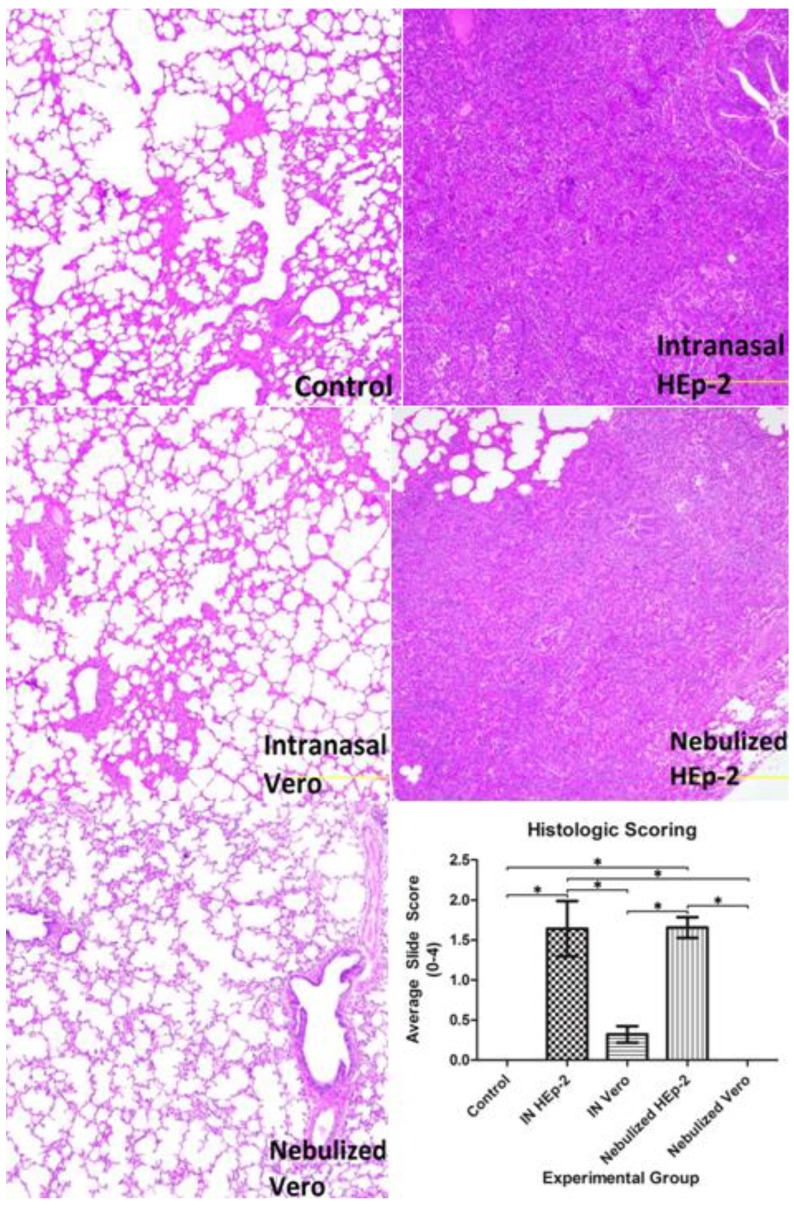
Histopathologic findings of selected lung lesions from each group. Lung from a control lamb with normal histologic appearance (Control). Lung from a lamb with intranasal inoculation of HEp-2-grown virus (INH) with dense consolidation around neutrophilic bronchiolitis (Intranasal HEp-2). Lung from a lamb with intranasal inoculation of Vero-grown virus (INV) with slight thickening of bronchiolar adventitia and minimal alveolar septal thickening both caused by infiltrate of lymphocytes and macrophages (Intranasal Vero). Lung from a lamb with nebulized RSV grown in HEp-2 (NeH) with a consolidation pattern similar to INH, although fewer areas are consolidated (Nebulized HEp-2). Lung from a lamb with nebulized RSV grown in Vero cells (NeV) that lacks lesions (Nebulized Vero). The INH group had a significantly higher score (see methods for scoring criteria) than control, INV and NeV groups, while NeH had a significantly higher score than control, INV, or NeV groups, *p* < 0.05.

Intranasally-inoculated lambs with gross lesions had multifocal to confluent foci of cellular infiltrate that filled bronchioles and alveoli and expanded the alveolar interstitium consistent with RSV infection reported previously in lambs [5,9,10]. This infiltrate was composed of lymphocytes, neutrophils, macrophages, and plasma cells. Bronchioles and alveoli contained moderate to abundant amounts of sloughed, degenerate epithelial cells and neutrophils. Large epithelial syncytial cells were present within bronchioles. Pulmonary epithelium lining affected airways was low to plump cuboidal with oval, vesiculate nuclei (type II pneumocyte hyperplasia). The INH group tended to have smaller, more dispersed lesions with a similar cellular population, and presence of fewer epithelial syncytia. Scoring of the histologic sections yielded a significantly higher score in NeH than the control or either of the Vero-inoculated groups, and INH lambs had a significantly higher score than the control or either of the Vero-inoculated groups (Figure 4). Lambs inoculated with Vero-grown virus and euthanized at 3 and 14 days p.i. lacked significant lesions.

### 3.4. Immunohistochemical Detection of RSV Antigen

**Figure 5:**
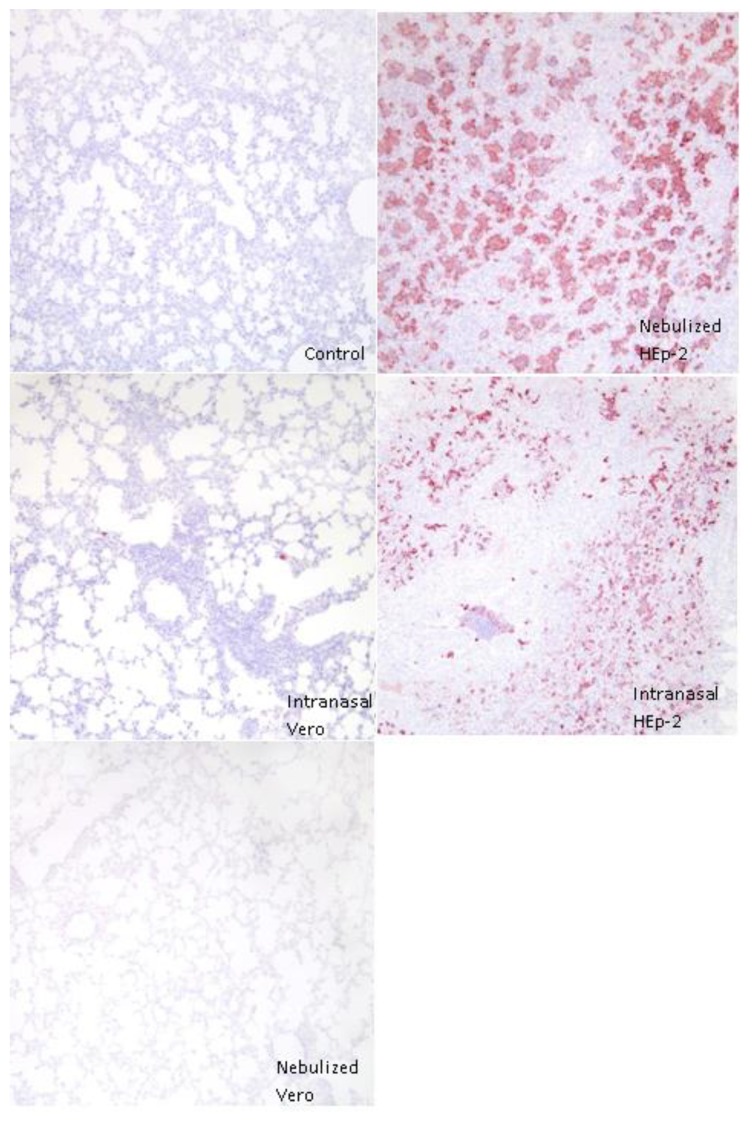
Immunohistochemical staining for RSV antigen. Representative immunohistochemical staining of lambs inoculated with respiratory syncytial virus (RSV), 10X magnification. Viral antigen is widely dispersed and staining is intense in lung of lambs infected with RSV grown in HEp-2 cells while lambs inoculated with Vero cell grown RSV have minimal viral antigen.

**Figure 6:**
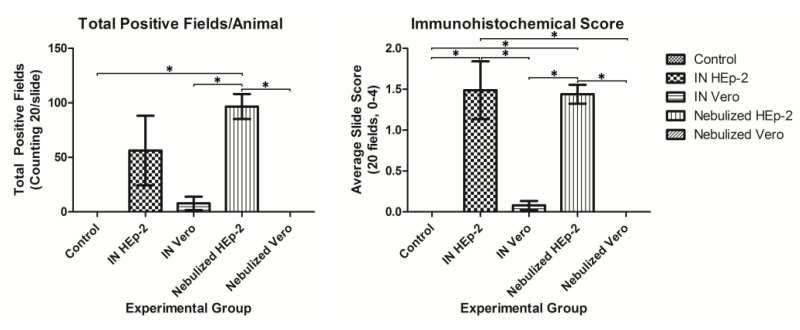
RSV immunohistochemical stain scoring. Seven slides with two sections each of lung lobe (at least 1 cm × 1 cm) were scored for staining of RSV antigen. Slides were assessed for number of cells per 10X field with RSV antigen staining which was then given a score according to the following scale: 0 = 0, 1 = 1–10, 2 = 11–39, 3 = 40–99, 4 =>100; slides were also assessed for the number of 10X fields with RSV antigen out of 20 fields for each slide. Both groups inoculated with HEp-2-grown RSV had higher average slide scores than control or either group inoculated with Vero-grown RSV. Nebulized HEp-2-grown RSV had significantly more fields with RSV antigen than control or Vero-grown RSV groups, *p* < 0.05.

Control lambs and NeV lambs lacked staining for RSV antigen while other inoculated lambs had positive cells present in or near areas of consolidation (Figure 5). Antigen was present in epithelial cells lining alveoli, bronchioles and within the cytoplasm of occasional macrophages. Scoring of the histologic sections yielded a higher overall score per animal in INH compared to control or INV groups, and a higher overall score in NeH versus control or INV groups (Figure 5). The NeH group had a higher number of total positive fields per animal compared to control or either Vero-infected group (Figure 6). Lambs inoculated with Vero-grown virus and euthanized at 3 and 14 days p.i. lacked significant viral antigen.

### 3.5. RSV and Cytokine mRNA Levels

**Figure 7:**
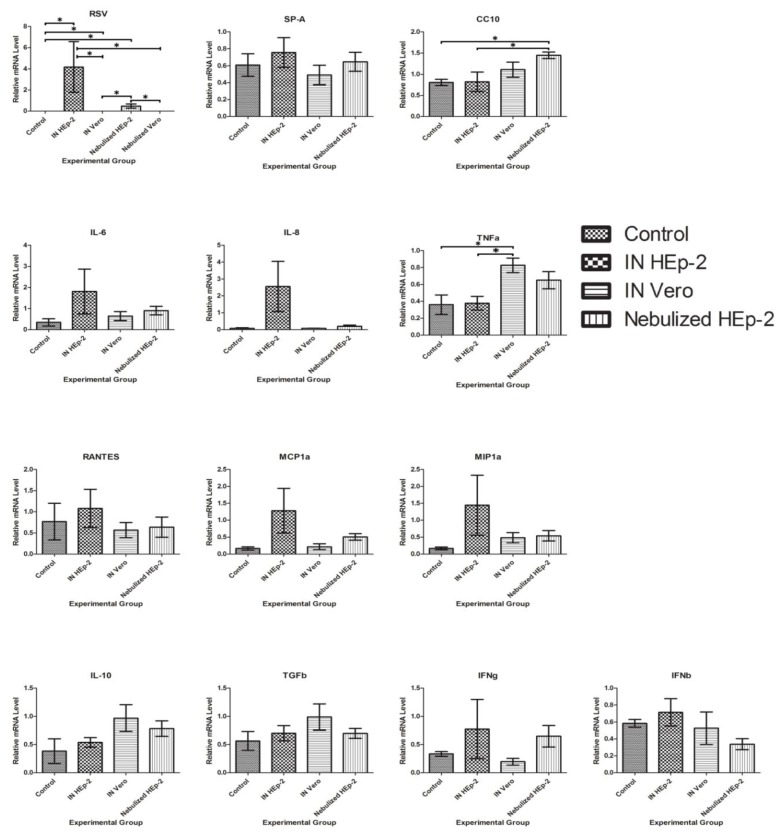
Relative (RT-qPCR) mRNA levels of respiratory syncytial virus (RSV) and immune and inflammatory mediator targets, relative to standard curve. Significant differences between groups (*p* < 0.05) are represented by a (*). Clara cell secretory protein (CC10), surfactant protein A (SP-A), interleukin 6 (IL-6), interleukin 8 (IL-8), interleukin 10 (IL-10), macrophage inflammatory protein (MIP-1α), monocyte chemotactic protein (MCP1α), tumor necrosis factor alpha (TNF-α), transforming growth factor beta (TGF-β), interferon beta (IFN-β), interferon gamma (IFN-γ), and regulated on activation normal T cell expressed and secreted (RANTES).

Lung homogenates from each animal were assessed by RT-qPCR for relative mRNA levels of M37 RSV (M37). Control, INV, INH, and NEH tissues were also assessed for Clara cell secretory protein (CC10), surfactant protein A (SP-A), interleukin 6 (IL-6), interleukin 8 (IL-8), interleukin 10 (IL-10), macrophage inflammatory protein (MIP-1α), monocyte chemotactic protein (MCP-1α), tumor necrosis factor alpha (TNF-α), transforming growth factor beta (TGF-β), interferon beta (IFN-β), interferon gamma (IFN-γ), and regulated on activation, normal T cell expressed and secreted (RANTES). NeV tissues were not assessed for additional targets at the time of initial study and tissues were not available for retrospective analysis. Control animals and both NeV lambs lacked expression of RSV RNA by RT-qPCR while significant differences in viral levels were present between all groups except the two HEp-2-inoculated groups. CC10 levels were significant higher in NeH than either control of INH groups. TNF-α was significantly higher in INV lambs than either control or INH groups (Figure 7). Lambs inoculated with Vero-grown virus and euthanized at 3 and 14 days p.i. had minimal viral antigen at day 3 but less than the NeV lambs and no viral antigen at day 14 (data not shown).

## 4. Discussion

HEp-2-grown M37 virus caused disease when inoculated intranasally or when nebulized in neonatal lambs. In contrast, Vero-grown virus caused minimal clinical disease. Additionally, lower levels of viral RNA were detected in intranasal Vero (INV) than in either method of inoculation with HEp-2-grown virus. Although this is a limited study, the *in vivo* results, demonstrating differences in viral infectivity, are consistent with the *in vitro* mechanistic studies demonstrating differences in the G glycoprotein expression by RSV grown in Vero cells, that were confirmed in data shown in the present study, and which can result in decreased attachment of the virus to epithelial cells [13,14,15,16,17,18,19,20,21,22,23,24,27,28,29].

The nebulized Vero (NeV) lambs had lower respiratory rates than nebulized HEp-2 (NeH) lambs on the day of inoculation and the INV lambs on days 3 and 6, but had no pathology or viral RNA detected post-mortem. Thus, their lower respiratory rate is attributed to natural variation. The infected lambs that differed from the NeV lambs did not have respiratory rates significantly above those of the control group and none of the groups’ average respiratory rate was elevated or decreased significantly over the course of the study. Infection with the M37 strain appears to typically cause an increased expiratory effort and may either increase or decrease respiratory rate; causing tachypnea with less expiratory effort and slowing respirations with greater effort. Coughing by inoculated lambs is rare, but wheezing will sometimes accompany the expiratory effort, though it is usually mild and detectable only with a stethoscope. On day 6 p.i., the increased expiratory effort in the INH was significantly higher than control lambs, which was consistent with a previous experiment using M37 (manuscript in preparation). The lack of significant alterations at days 3 and 14 p.i. in lambs nebulized with Vero-grown virus demonstrates that viral replication did not peak earlier or later than the six day time point.

TNF-α is an acute-phase, proinflammatory cytokine that has been shown to be increased in nasopharyngeal secretions at the time of hospitalization for RSV infection in infants [30]. In a previous study, we observed that an increase in TNF-α was shown to occur in inoculated lambs at day 3, but returned to control levels by day 6 p.i. [31]. Thus, increased TNF-α in the INV group is likely not due to RSV infection but perhaps a secondary underlying bacterial infection in some of the lambs since the lambs are acquired colostrum-deprived at 1–2 days of age. The variation in CC10 levels is difficult to interpret, as initial death of Clara cells will release CC10 or CC10 can be upregulated, while a reduction in Clara cells decreases overall production of CC10. Additionally, Clara cell responses tend to be localized, so sampling can affect results as well. None-the-less, the increase in CC10 in the NeH group is intriguing. Further characterization of Clara cells and CC10 in RSV throughout the course of disease as well as at the cellular level, possibly by laser capture microdissection techniques could be illuminating as to the role this cell type and protein play in RSV disease. Several other cytokines (IL-6, IL-8, RANTES, MCP-1α, MIP-1α, IFN-γ, and IFN-β) showed a trend of increased expression with intranasal inoculation of HEp-2-grown M37 and additional animals would likely clarify these trends.

An additional finding that may be important in further model development is the distribution of lesions with intranasal versus nebulized HEp-2-grown virus. Virus was detected by RT-qPCR in all nebulized lambs, but only 3 of 4 intranasally-inoculated lambs. Thus, while viral RNA levels and immunohistochemical scores were higher, and consolidation and histologic lesions more severe in the intranasally-inoculated lambs, more lambs had detectable viral RNA levels at 6 days p.i. in the nebulized group. Additionally, while a greater number of cells were positive by IHC in the intranasally-inoculated lambs, a greater portion of microscopic fields had detectable antigen in the nebulized lambs. The nebulization reduces viral titer by roughly one log (data not shown; manuscript under review) and thus may contribute to the decreased infectivity of nebulized inoculum; however, this likely affects both HEp-2 and Vero-grown virus similarly. Various types of nebulizers and types of nebulization delivery (increased volumes with various titers, longer duration) may further affect the degree of disease severity. Our recent work demonstrates that nebulized delivery of RSV M37 grown in HEp-2 cells causes consistent disease with high titers in bronchoalveolar lavage fluid [30,31,32].

In summary, HEp-2-grown M37 hRSV administered by intranasal or nebulized methods causes increased disease in neonatal lambs (*in vivo*) when compared to Vero-grown virus and this is perhaps due, at least in part, to a truncated viral G attachment protein. Additionally, nebulized virus yields a different pattern of gross and histologic lesions and may yield more consistent infection. This study and others continue to demonstrate that sheep are a useful model of perinatal infection using intranasally-inoculated or nebulized HEp-2-grown RSV.
